# Estimating the incidence of cocaine use and mortality with music lyrics about cocaine

**DOI:** 10.1038/s41746-021-00448-x

**Published:** 2021-06-30

**Authors:** Yulin Hswen, Amanda Zhang, John S. Brownstein

**Affiliations:** 1 Department of Epidemiology and Biostatistics, University of California San Francisco, San Francisco, CA USA; 2 Bakar Computational Health Sciences Institute, University of California San Francisco, San Francisco, CA USA; 3 Innovation Program, Boston Children’s Hospital, Boston, MA USA; 4 Computational Epidemiology Lab, Harvard Medical School, Boston, MA USA; 5 Applied Mathematics, Harvard University, Cambridge, MA USA

**Keywords:** Communication, Risk factors

## Abstract

In the United States, cocaine use and mortality have surged in the past 5 years. Considering cocaine’s reputation as a fashionable social drug, the rise of cocaine mentions in popular music may provide a signal of epidemiological trends of cocaine use. We characterized the relationship between mentions of cocaine in song lyrics and incidence of cocaine use and mortality in the US. Incidence of cocaine use from 2002 to 2017 was obtained from the National Survey on Drug Use and Health and cocaine overdose mortality rate from 2000 to 2017 was obtained from the Centers for Disease Control. Distributed lag models were fit using ordinary least squares on the first difference to identify associations between changes in cocaine lyric mentions and changes in incidence of cocaine use and mortality. A total of 5955 song lyrics with cocaine mentions were obtained from Lyrics.com. Cocaine mentions in song lyrics were stable from 2000 to 2010 then increased by 190% from 2010 to 2017. The first-order distributed lag model estimated that a 0.01 increase in mentions of cocaine in song lyrics is associated with an 11% increase in incidence of cocaine use within the same year and a 14% increase in cocaine mortality with a 2-year lag. Lag-times were confirmed with cross-correlation analyses and the association remained after accounting for street pricing of cocaine. Mentions of cocaine in song lyrics are associated with the rise of incidence of cocaine use and cocaine overdose mortality. Popular music trends are a potentially valuable tool for understanding cocaine epidemiology trends.

## Introduction

Cocaine popularity in the United States has rebounded in the past 5 years after many years of decline. Annual deaths by cocaine increased 200% from 2012 to 2017, making cocaine the leading non-opioid cause of drug overdose death in the US^1^. At the same time, cocaine’s user base has expanded with more people trying cocaine for the first time. From 2012 to 2017, the number of people using cocaine for the first time in the past year increased by 57%^2,3^. In 2017, 6.2% of people in the US aged 18–25 reported using cocaine in the past year, compared to 0.6% for heroin^3^.

Historically, patterns of cocaine use differed from those of other illicit drugs in that cocaine has been used almost exclusively in social settings, especially to indicate high social status. Cocaine possesses the unique reputation of being the “rich man’s speed,” a symbol of affluence flouted by celebrities and artists and the object of aspiration for others^4^. The glamorization of cocaine’s bodily effects and an underestimation of its dangers likely also influenced public perception of cocaine. A 1977 Newsweek story on cocaine reported “Among hostesses in the smart sets of Los Angeles and New York, a little cocaine, like Dom Perignon and Beluga caviar, is now de rigueur at dinners”^5^. Although the impression of cocaine is that it gives the user strength and vigor, its actual use is harmful. In a study ranking the danger of drugs^6^, cocaine received the highest harm ratings and notably scored highest in the “social harm” category. Furthermore, epidemiological evidence on cocaine has shown it to be a highly addictive and widely abused drug specifically among young people^6,7^.

*“In its pharmacological action, cocaine—perhaps more than any other of the recognized psychoactive drugs—reinforces and boosts what we recognize as the highest aspirations of American initiative, energy, frenetic achievement, and ebullient optimism…*”*—Stimmel, 1984*^4^

Its attractiveness among the public has been arbitrated by popular media, including coverage of celebrity drug activities associating the drug with high society^2^. In these ways, references to cocaine in the media and popular culture portrayed real-world behaviors of cocaine use.

There is reason to believe that popular culture today is again capturing real-world trends around a renewed interest in cocaine. Although cocaine’s reputation waned during the 1980s War on Drugs government crackdown, other forms of popular media such as the music industry continue to relay information about cocaine. Major artists today frequently mention cocaine and other drugs extensively in their songs, particularly in the context of glamour, wealth, and sociability^8,9^. For instance, studies analyzing popular music and videos have found drugs to be a dominant theme^8,10–12^ with one study reporting that 33.3% of top-charting songs portrayed substance use with an average of 32.5 drug references per hour^9^. Considering that 90 percent of Americans regularly listen to music with an average listening time of 32.1 h per week^11^, this means Americans on average are exposed to 54 explicit references to drugs every day.

A growing body of research supports that drug mentions in popular media may be linked with substance use, including for smoking^13–15^, alcohol^16^, and cannabis^17^. A qualitative study found that an underground form of hip-hop music called “screw” is a strong reinforcer of codeine syrup use; respondents named “media modeling” as the foremost reason for the popularity for syrup usage^18^. A content analysis of another genre of music, popular country music, found that lyrics were more likely to describe women in association with alcohol use and sex in the 2010’s compared to earlier decades, confirming the known association between alcohol use and sexual assault in this genre^19^. However, the majority of these studies on substance use in song lyrics have been descriptive and limited in the scope of songs analyzed. For instance, Primack et al.^9^ conducted a content analysis of 279 songs for drug-related content in the single year of 2005 to determine degree of drug exposure for music listeners. Hall, West, and Neeley^20^ analyzed time trends of alcohol, tobacco, and other drugs in 1100 songs over 50 years, an average of only 22 songs per year. Herd performed a qualitative analysis of the social context surrounding 341 rap songs^8^. While these studies describe important findings regarding drug exposure in song lyrics, trends over time, and qualitative content analysis, none were specific to cocaine nor quantified the effect size of drug exposure in song lyrics with drug use behavior. Additionally, all studies compiled song titles from ranked lists, most commonly the Billboard Top-100, thereby skipping over thousands of popular songs that did not make the list.

Given cocaine’s fashionable reputation and highly social patterns of use^21^, descriptions of cocaine in music are potentially an indication of increased cocaine initiation and use. However, the epidemiological trends between popular media and cocaine have yet to be studied empirically. This study investigated the relationship between prevalence of drug mentions in music lyrics and epidemiological trends for cocaine. We sought to offer insights on the recent rise in cocaine use incidence and cocaine overdose mortality observed during the first decades of the 21st century with respect to the influence of contemporary music trends on population-based health behaviors.

## Results

### Descriptive results

**Fig. 1:**
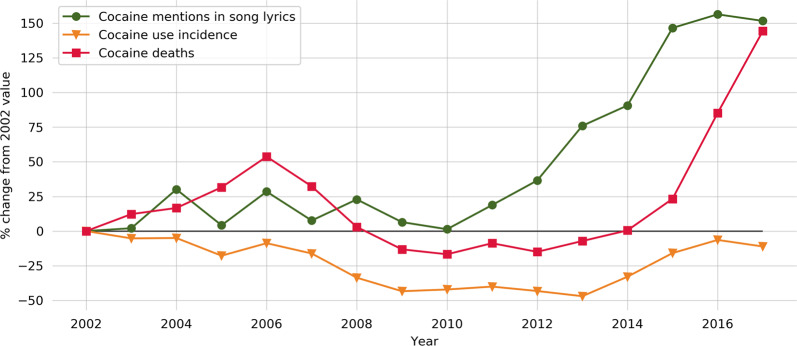
Cocaine mentions in song lyrics, incidence of cocaine use, and cocaine death rate. Summarizes the percent change from the baseline year of 2002 for 2002-2016 for cocaine mentions in song lyrics, incidence of cocaine use, and cocaine death rate.

A total of 6923 unique mentions of cocaine from 5955 unique songs and 2418 unique artists were obtained from Lyrics.com. The mean number of cocaine mentions per year was 364 (SD = 73). Cocaine mentions in lyrics was stable from 2000 to 2010, then increased by 190% from 2010 to 2017. Data on cocaine overdose mortality, obtained from the Centers for Disease Control, also exhibited a recent surge. Deaths increased every year starting in 2012 with a total growth of 200% from 2012 to 2017. The percent change in cocaine mentions in song lyrics, incidence of cocaine use from 2002 for 2002-2016 is visualized in Fig. 1.

The top three cocaine slang words used from 2000 to 2018 were “coke”, “cocaine”, and “powder” and made up over 60% of drug mentions in song lyrics. “Coke” led with 2358 song counts (34%), then “cocaine” with 1469 song counts (21%), “powder” with 810 song counts (12%), “coca” with 579 song counts (8.4%) and “kilo” with 470 song counts (6.8%). Slang terms with the greatest growth from 2000 to 2018 were “kilo” at 1950%, “yayo” at 750%, and “coco” at 640%, followed by “baking soda” at 300%.

### Distributed lag regression

Results from the time distributed-lag model are presented in Table 1 and show that a relative 0.01 increase in mentions of cocaine relative to “love” mentions in song lyrics was associated with a 100 * (e^(0.01*11.1609) − 1) = 11.8% increase in incidence of cocaine use during the same year and a 100 * (e^(0.01*14.398) − 1) = 15.5% increase in mortality by cocaine in 2 years. These associations were robust to changes in street price. A decrease in cocaine price was associated with an increase in cocaine mortality with a lag of 2 years.

**Table 1:** Parameter estimates for distributed lag models.

	Death model	Death model	Use model	Use model
	*β*	*p*	*β*	*p*
Intercept	0.42	0.142	0.08	0.752
Lyrics (same year)	–	–	11.16	0.039
Lyrics (1 year ago)	9.48	0.162	6.22	0.193
Lyrics (2 years ago)	14.39	0.037	–	–
Lyrics (3 years ago)	7.35	0.331	–	–
Price (same year)	−0.00	0.118	−0.00	0.573

Lag-time results were robust to sensitivity analyses that removed the term “8-ball” (death model: *p* = 0.04, cocaine use model: *p* = 0.03). No significant lag-times were seen for the distributed lag regression for the control models of codeine lyrics and heroin lyrics with cocaine epidemiology.

**Fig. 2:**
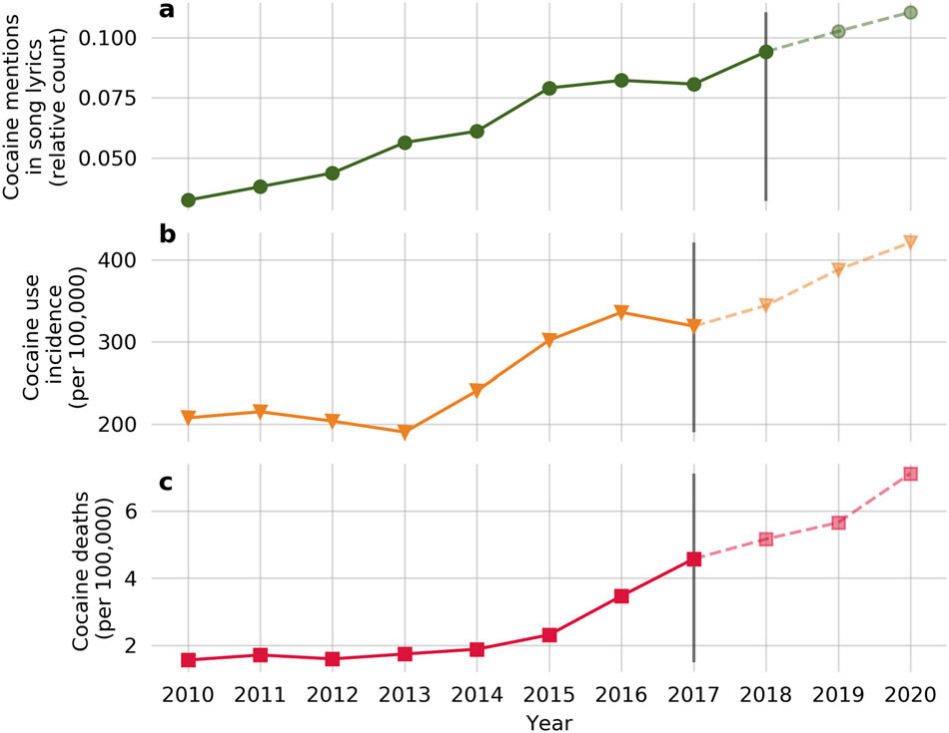
Cocaine use and cocaine-related death rates for 2018, 2019, and 2020. **a** Projection of cocaine mentions in song lyrics for 2019 and 2020. **b** Projection of cocaine use incidence for 2018, 2019, and 2020. **c** Projection of cocaine mortality for 2018, 2019, and 2020. Predictive models were fitted with lyrics and epidemiological data from 2000 to 2018 and assumed linear growth of cocaine mentions in song lyrics.

Regression parameter estimates were used to project future incidence of cocaine use and cocaine-related death rates for 2018, 2019, and 2020 and these results are presented in Fig. 2. Based on the model, we estimate the incidence of cocaine use and cocaine overdose mortality will continue to increase. We project incidence of cocaine use to be 344 new users per 100,000 in 2018, 385 new users per 100,000 in 2019, and 416 new users per 100,000 in 2020. We project overdose deaths by cocaine to be 5.16 deaths per 100,000 in 2018, 5.66 deaths per 100,000 in 2019, and 7.12 deaths per 100,000 in 2020.

### Cross-correlation

**Fig. 3:**
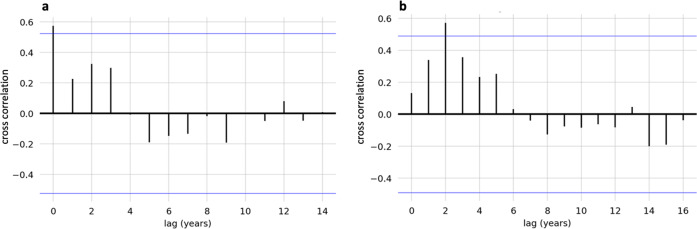
Cross-correlation between cocaine lyrics and incidence of cocaine use. **a** Cross correlation for cocaine mentions in lyrics leading cocaine use incidence at different lag times. **b** Cross correlation for cocaine mentions in lyrics leading cocaine deaths at different lag times. Blue lines indicate the 95% confidence interval around cross-correlation value of 0.

Cross-correlation analyses are displayed in cross-correlation plots in Fig. 3. These analyses confirmed a significant cross-correlation between cocaine lyrics and incidence of cocaine use in the same year (*r* = 0.5713, *p* < 0.05) and a significant correlation between cocaine lyrics and mortality 2 years later (*r* = 0.5747, *p* < 0.05). Sensitivity analyses did not yield significant cross-correlations in the lags in the opposite direction. Cocaine mortality was not associated with future cocaine mentions in lyrics at any lag time.

## Discussion

Our study explores the ability of song lyrics to signal epidemiological trends in incidence and mortality of cocaine use. Specifically, our results found that an increase in cocaine mentions in song lyrics is associated with increased incidence of cocaine use in the same year and death by cocaine 2 years later. The temporal relationship between cocaine lyrics and incidence of cocaine use and related deaths was confirmed with significant cross-correlation in the same year for cocaine use and 2 years for cocaine overdose mortality. The lead time period of 2 years found in this study between lyrics mentioning cocaine and incidence of cocaine and cocaine overdose mortality is supported by epidemiological evidence that the time between initiation of cocaine use and seeking help for addiction is between 1 and 3 years^22,23^. Musical trends that depict cocaine in lyrics may be an early signal of a rising interest and use of cocaine in the same year. The lag-time period of 2 years between cocaine lyrics and cocaine mortality may indicate that there is an incubation period of 2 years between addiction and fatality. Treatment intervention is critical in this time period to prevent the onset of drug dependence and death.

Our results also provide insights into the pathways by which media may influence cocaine use behaviors. In addition to being a measurement tool to estimate cocaine epidemiology, media itself can influence public perceptions of drug use and lead to increased drug use at the population level. Therefore, music about cocaine may not only provide an early signal of cocaine-related behaviors, but it could also act as an exposure that encourages the use of cocaine. Our study did not test the potential dynamic and cyclical relationship between cocaine lyrics and patterns of cocaine use. While cocaine mortality did not appear to estimate future cocaine mentions in song lyrics, further investigation into the influence of cocaine use on song lyrics is warranted.

Our study showed growth in slang terminology such as “kilo”, “yayo”, “coco”, and “baking soda” instead of more explicit terms for cocaine such as “coke” and “cocaine” used in older song lyrics. These results may indicate that the current generation has inherited an attachment to cocaine expressed through the development of new slang terms for cocaine. Evidence of this shifting use of cocaine by younger generations is provided by a 2018 United Nations study reporting that settings of cocaine use have branched out from exclusive clubs to include more accessible environments including college parties, concerts, and bars^22^. Furthermore, in the past decade, first time users of cocaine have moved from high income areas to lower income and under resourced neighborhoods^24^. The popularity of new slang terms in our study may signal a different population and a reemergence of a black market for cocaine as a rebranding of street terminology is being used to disguise discussion of this illegal drug. Finally, our argument about epidemiological ties with cocaine mentions in song lyrics is strengthened by our results that showed that popular music lyrics related to codeine and heroin did not follow the same trends with cocaine mortality. If this trend in cocaine lyrics continues, we estimate that future incidence of cocaine use may increase by close to 40% and mortality by cocaine use will increase by almost 80% for 2020, making it important to monitor this growth in lyrics about cocaine over time.

An important limitation with our study is that our results depict associations and cannot confirm causality. Analytical steps were included in the methodology to remove potential spurious correlations but this is not enough to deem our results as causal. Therefore, our findings should be interpreted with caution. Cocaine street price was included in the model to control for economic fluctuations that would impact purchasing behaviors and initiation of drug use^25^. Spurious correlations were controlled through first-order differentiation. Further tests on the differenced series confirmed autocorrelations were no longer present after first-order differentiation.

Another limitation of our study is the potential of not having captured all the cocaine slang terminology to identify song lyrics describing cocaine. Although we queried a number of top websites for the most referenced slang terms for cocaine, it is possible that certain ambiguous slang terms were excluded. Generating a comprehensive list of slang drug terminology is exceedingly difficult because the vocabulary around cocaine evolves to conceal its discussion^26^. However, sensitivity analyses revealed that our results were robust to the removal of ambiguous terms such as “8-ball”. Inclusion of additional terms like “blow”, which was not included in the model because of its possible description of guns rather than drugs, increased the degree of association between mentions in lyrics and cocaine mortality. Because of this, we believe that genuinely uncertain references to cocaine are rare and have limited potential to significantly alter study results.

In this study, we were only able to conduct analyses at the yearly level and not more granularly. Data derived from Lyrics.com is only available at the annual level. We explored several song lyrics engines including Spotify, Genius Lyrics, Metro lyrics, and Billboard; however, these other platforms either do not allow their data to be available or it is also at the yearly level. We selected Lyrics.com because of its word query capabilities within lyrics that other song lyric engines do not provide. This restricted us to have 18 effective observations from 2000 to 2017 for song lyrics data. However, in our analysis, another limiting factor was the incidence of cocaine data from the National Survey on Drug Use and Health published by the Substance Abuse and Mental Health Services Administration and cocaine-related deaths were obtained from the Center for Disease Control Multiple Causes of Death WONDER database, which only are available aggregated at the yearly level. Therefore, our analysis still would have been limited to this level of granularity. Additionally, since our definition of cocaine-related mortality includes deaths using the ICD-10 code T40.5, it may include underlying causes of death other than cocaine. However, other ICD-10 codes in the mortality dataset do not specify the actual drug involved. While this is a limitation in the use of death certificates for identifying cause-of-death, this is a consistently used measure for cocaine-related deaths. Furthermore, the quality of testing for drug overdose has improved over time^27^. Thus the rise in deaths by cocaine may be in part due to the more accurate determination of deaths attributed to cocaine.

Lastly, it is possible that mortality by cocaine could occur in the same year of initiation, which was not seen in our results. However, addiction to cocaine is often characterized by repeated use that changes brain and psychological function that promote transitions to problematic patterns of use^28^. Thus, the accumulation of conditional use of cocaine overtime that leads to mortality by cocaine is likely to occur after years of addictive use of cocaine.

In conclusion, these associations underscore the importance of monitoring trends in music to understand drug patterns over time. Media are a powerful indicator of social norms and our study offers initial epidemiological evidence that music lyrics about cocaine may provide an early signal to incidence of cocaine use and mortality. Additionally, new slang terminology for drugs in music lyrics could indicate a new generation of cocaine users and a surge in under-detected use. Future studies should conduct a deeper investigation into lyrics about cocaine and other substances, with particular focus on how these messages may shape cultural perceptions and behaviors toward drug use. Given the wide audience of popular music, popular music artists should consider the potential influence of their lyrics on the drug epidemic impacting today’s youth.

## Methods

### Data

The use of computers to record, distribute, store and play music in the early 2000’s shifted the way people accessed and listened to music^29^. The digital transformation of the music industry beginning in the early 2000’s has allowed peer-to-peer sharing of digital audio files, as well as decreased the barriers to entry for artists^30^. For instance, artists can now self-release their music to various online platforms, thereby decreasing the time required for a song to be written, performed, and released to the public, and making it possible for emerging artists to expand the reach of their audience^31,32^. Additionally, the most recent surge of cocaine use and deaths occurred in the 2010s^33^. Therefore, our data was restricted to 2000-onward to focus on contemporary trends in music and the most recent resurgence of cocaine use and mortality in the United States.

### National incidence of cocaine use

Data on incidence of cocaine use from 2002 to 2017, defined as the nationwide number of new cocaine initiates aged 12 and older in the past year, was obtained from Table 7.28A of the 2017 National Survey on Drug Use and Health published by the Substance Abuse and Mental Health Services Administration^3^. National incidence was calculated by dividing initiation counts by midyear population estimates obtained from the United States Census Bureau^34^.

### National drug overdose deaths involving cocaine

National drug overdose deaths involving cocaine were collected as yearly counts of cocaine-related deaths for 2000–2017, obtained from the Center for Disease Control Multiple Causes of Death WONDER database^1^. Query criteria for drug overdose involving cocaine were all conditions under the ICD-10 code T40.5 (“Poisoning by, adverse effect of and underdosing of cocaine”). National death count by cocaine was divided by midyear population estimates obtained from the United States Census Bureau^34^.

### Cocaine mentions in song lyrics

To measure cocaine mentions in song lyrics, first a comprehensive list of informal words that describe cocaine (slang) was compiled by analyzing top websites that have the primary purpose of deciphering slang terminology such as Urban Dictionary^35^ as well as the Drug Enforcement Association Slang Terms and Code Words reference guide^36^. Two independent researchers coded a random sample of 1000 to remove ambiguous terms that did not refer to cocaine. Slang terms such as “snow”, “blow”, and “dust” were found to be used in reference to connotations other than cocaine in the lyrics’ dataset, and were therefore excluded from the set of terms. The final list used for this analysis was composed of 18 terms: “cocaine”, “coke”, “coca”, “coco”, “powder”, “baking soda”, “bakin soda”, “arm and hammer”, “kilo”, “8 ball”, “eight ball”, “llello”, “yayo”, “yeyo”, “nose candy”, “white horse”, “white line”, and “doing lines”.

### Cocaine time trends

Yearly counts of cocaine mentions in song lyrics were obtained from Lyrics.com, the largest known database for references to song lyrical information with keyword search^37^. A programmatic code was written to query each individual slang term to obtain the count of songs that mentioned that term each year. Slang term counts were summed to obtain the total mentions of unique slang terms per year. To account for variation in song volume over time, we transformed counts of cocaine mentions into a ratio of relative prevalence by dividing mentions of cocaine terms by mentions of “love”. Previous studies have shown that love is consistently the dominant theme in song lyrics for every decade since the 1960s^38,39^, therefore “love” was used in this study to adjust for changes in song volume over time and provide a relativistic interpretation for the time trends.

### Cocaine street price

Cocaine street price data in the US from 2000 to 2016, adjusted for purity and inflation, was obtained from the United Nations Office on Drugs and Crime^26^.

### Analysis

To examine the association of cocaine mentions in lyrics with incidence of cocaine and mortality, we used a first-order finite distributed lag model with unrestricted coefficients. In this model, the first differences of the time series of national counts of incidence of cocaine and mortality were regressed on first differences of the times series of cocaine mentions in lyrics earlier in time^27,40–43^.

### First-order differencing

To regress time-series data, the data must be stationary whereby the mean and variance are independent of time. To test the stationarity of our data, we conducted Augmented Dickey–Fuller (ADF) tests on the lyrics, price, incidence, and mortality time series. ADF tests deemed the data was nonstationary and a time trend in the data existed. To transform our data to be stationary, we differenced the data on the first order. Inspection of autocorrelation function plots and partial autocorrelation function plots before and after first order differencing confirmed autocorrelation was removed after differencing. Subsequent analyses were performed using the differenced series.

### Distributed lag regression

A standard epidemiological method for identifying relationships between time series is distributed lag regression^43,44^. The general format of this model is presented in Eq. (1). In this framework, the dependent variable is regressed on delayed (or lagged) instances of the independent variable. This practice is commonly used in economics^42^ and has been applied to epidemiological research^41^ in order to express associations that change over time^43^. In our study, the dependent variable was cocaine mortality or cocaine use incidence and the independent variable was cocaine mentions in song lyrics. We chose regression terms using a stepwise process, beginning with contemporary values and adding previous year lag terms one at a time until further additions were no longer significant^40^. Epidemiological research and theory support a 1–3 year delay between media exposure and behavior^22,23^. Thus only lag years were included in the model that were consistent with a potential epidemiological relationship. For instance, we did not include year 1 in the distributed lag model because of the lack of biological plausibility that music about cocaine would estimate cocaine overdose mortality in the same year. The final regression model for cocaine mortality is presented as Eq. (2) and the final regression model for cocaine use incidence is presented as Eq. (3).

Sensitivity analyses were conducted whereby the term “8-ball” was excluded—although most frequently used to reference cocaine in music lyrics^31^, “8-ball” can also be used as a reference to crack cocaine. Additionally, we employed a negative control by collecting slang terms for codeine: ‘codeine’, ‘purple drank’, ‘sizzurp’, ‘syrup’, ‘lean’; and for heroin: ‘heroin’, ‘skag’, ‘china white”,’ white horse’, ‘black tar’, ‘black eagle’, ‘brown sugar.’ These sets of terms were tested in the distributed lag model in the same procedure to determine if there was a relationship between cocaine epidemiology and drug lyrics about non-cocaine drugs.

The models were fit using ordinary least squares and included variables that could act as “proxy” confounders—those that may have distorted the relationship between lyrics and mortality^45^. The response variable was logged to interpret the regression coefficients as percent changes. The fitted model and 2018 lyrics data about cocaine were used to project 2018, 2019, and 2020 incidence of cocaine and cocaine overdose mortality assuming a linear rate of growth of cocaine prevalence in song lyrics.

#### Model

1 $${{\Delta }}\ln \left( {{\mathrm{y}}_{\mathrm{t}}} \right) = {\upalpha} + {\upbeta}_0{{\Delta }}x_t + \beta _1{{\Delta }}x_{t - 1} + \ldots + \beta _p{{\Delta }}x_{t - p} + {\it{\epsilon }}_{t,}$$

where Δ*x* = *x*_*t*_ − *x*_*t−*1_

2 $$\Delta \ln \left( {deaths_t} \right) = \alpha + \beta _1{{\Delta }}lyrics_{t - 1} + \beta _2{{\Delta }}lyrics_{t - 2} + \beta _3{{\Delta }}lyrics_{t - 3} + \beta _4price_t + {\it{\epsilon }}_t$$

3 $$\Delta \ln \left( {use_t} \right) = \alpha + \beta _0\Delta lyrics_t + \beta _1\Delta lyrics_{t - 1} + \beta _2price_t + {\it{\epsilon }}_t$$

### Cross-correlation

To provide further validation of our lag-time results, we performed cross-correlation tests between lyrics and cocaine use and lyrics and deaths at varying lags to evaluate time-delayed associations. Cross-correlation analyzes the relationship between two signals and calculates the correlation coefficient at designated lags (displacements) between the two series. The correlation coefficient is maximal at the time point that the two series correlate most closely and the coefficient reveals the strength of the correlation. Cross-correlation analyses were conducted in both directions to examine whether the data supported cocaine epidemiology preceding lyrics or lyrics preceding cocaine epidemiology.

The cross-correlation between two discrete sequences *x* and *y* at lag *k* is defined as:

$$\left( {x \star y} \right)\left[ k \right] \buildrel \Delta \over = {{\Sigma }}_nx\left[ {n + k} \right] \cdot y^ \ast \left[ n \right],$$

where y*[n] denotes the conjugate of y.

All analyses were conducted using the statsmodels package in Python^46^.

### Reporting summary

Further information on research design is available in the Nature Research Reporting Summary linked to this article.

## Supplementary information

Reporting Summary Checklist

## Data Availability

The data that support the finding from this study are publicly available from The Substance Abuse and Mental Health Services Administration and The Centers for Disease Control and Lyrics.com. Aggregated datasets are available on reasonable request.

## Code availability

Statistical analyses were conducted using the statsmodels package in Python.
